# Reduced membrane attack complex formation in umbilical cord blood during Eculizumab treatment of the mother: a case report

**DOI:** 10.1186/s12882-019-1469-9

**Published:** 2019-08-07

**Authors:** Subagini Nagarajah, Martin Tepel, Christian Nielsen, Kristian Assing, Yaseelan Palarasah, Lise Lotte Torvin Andersen, Lotte Borg Lange, Claus Bistrup

**Affiliations:** 1 Department of Nephrology, Odense University Hospital, 5000 Odense C, Denmark; 2 Institute of Molecular Medicine, University of Southern Denmark, Odense, Denmark; 3 Department of Clinical Research, University of Southern Denmark, Odense, Denmark; 4 Department of Immunology, Odense University Hospital, Odense, Denmark; 5 Research Unit of Immunology and Microbiology, University of Southern Denmark, Odense, Denmark; 6 Department of Obstetrics and Gynecology, Odense University Hospital, Odense, Denmark

**Keywords:** Atypical hemolytic uremic syndrome, End-stage renal disease, Pregnancy, Complement, Eculizumab, Umbilical cord vein blood, Membrane-attack complex

## Abstract

**Background:**

Atypical hemolytic uremic syndrome (aHUS) is a disorder of the microvasculature with hemolytic anemia, thrombocytopenia and acute kidney injury. Nowadays, aHUS is successfully treated with eculizumab, a humanized, chimeric IgG2/4 kappa antibody, which binds human complement C5 and blocks generation of C5a and membrane-attack-complex.

**Case presentation:**

A 25-year-old woman with end stage renal disease due to relapsing atypical hemolytic uremic syndrome had a relapse of the disease during pregnancy. She was treated with eculizumab. We measured reduced formation of the membrane-attack complex in newborn’s umbilical cord vein blood using the sensitive and specific Palarasah-Nielsen-ELISA.

**Conclusions:**

Eculizumab treatment of the mother with end stage renal disease may cause reduced innate immunity which could render newborns more susceptible to infections.

## Background

Atypical hemolytic uremic syndrome (aHUS) is a disorder of the microvasculature with hemolytic anemia, thrombocytopenia and acute kidney injury. The pathogenesis of aHUS involves the uncontrolled activation of the alternative complement pathway [1–5]. Nowadays, aHUS is successfully treated with eculizumab [3–7]. Eculizumab is a humanized, chimeric IgG2/4 kappa antibody, which binds human complement C5 and blocks C5a generation and complement-mediated cell lysis via membrane-attack-complex [6]. However, it is not known whether the administration of eculizumab in pregnant patients with end-stage renal disease due to aHUS may cause reduced membrane-attack-complex formation also in the fetal circulation. The objective of the present study was to alert clinicians to the effect of therapeutic antibodies in newborns.

For this report we measured the deposition of complement C3 and C9 in the mother’s blood, in index newborn’s umbilical cord vein blood (obtained after delivery, i.e., 2 h after the last eculizumab infusion), and in blood 3 weeks after birth we measured deposition of complement C3 and C9 using the Palarasah-Nielsen-ELISA as previously described [8, 9]. The sensitive and specific Palarasah-Nielsen-ELISA determines the capacities of three complement pathways using wells pre-coated with immune complexes, lipopolysaccharides, or mannan, to activate classical, alternative, and lectin pathway, respectively [9]. The deposition of C3 was measured using monoclonal anti-human C3, clone C3 F1–8, which identifies C3, C3b, iC3b and C3c; and deposition of C9 was measured using anti-human C9 (Bioporto A/S, Gentofte, Denmark), which reacts with the membrane-attack-complex [9]. The advantages of the Palarasah-Nielsen-ELISA had been described [9]. Briefly, CH50 and AH50 methods are not based on ELISA principle but based on the spectrophotometric measurements of the degree of cell lysis following addition of antibody-sensitized sheep erythrocytes and sheep erythrocytes in solution, respectively. The protocol for the CH50 and AH50 methods is laborious, difficult to standardize, and it is well established that the ELISA methodology is more sensitive compared to these older techniques. Further, and in contrast to the CH50 and AH50 methods, Palarasah-Nielsen-ELISA is able to distinguish complement capacity between C3- and C9 (membrane-attack-complex) -level.

## Case presentation

### Clinical findings

A previously healthy 25-year-old woman presented to the hospital’s emergency department with high blood pressure, hemolytic anemia, thrombocytopenia, and oliguric acute kidney injury. Her blood pressure was 158/101 mmHg. Laboratory data revealed elevated plasma creatinine level, 925 μmol/L (normal range, 45–90), plasma urea, 34.1 mmol/L (normal range 2.6–6.4), reduced hemoglobin, 5.5 mmol/L (normal range, 7.3–9.5), plasma lactate dehydrogenase, 714 U/L (normal range, 105–205), reduced plasma haptoglobine levels less than 0.08 g/L (normal range, 0.35–1.85), and reduced platelet count, 42 per nL (normal range, 165–400). A peripheral blood smear showed 6–12 schistocytes per high power field (normal, less than 5). Antinuclear antibodies, antineutrophil cytoplasmic antibodies, anti-glomerular basement membrane antibodies, anti-complement factor H antibodies, and Hanta virus antibodies were negative. A Disintegrin And Metalloproteinase with a ThromboSpondin type 1 motif, member 13 activity was normal, thus excluding thrombotic thrombocytopenic purpura. Urine analyses showed microscopic hematuria and urinary protein/creatinine ratio was 3.807 mg/g.

Stool culture and multiplex polymerase chain reaction for verotoxin-producing *Escherichia coli* in stool were negative. A renal biopsy showed 7 glomeruli without fresh thrombotic material, but ischemic damage of glomeruli and tubuli. Vessels showed increased wall thickening without thrombotic material, which may indicate weak thrombotic microangiopathy, and immunofluorescence was negative.

### Genetic findings

Genetic workup revealed no mutations located in the genes for complement factor H, complement factor I, and membrane cofactor protein. The patient had a homozygous deletion of exon 3–6 in the complement factor H related gene 1 (CFHR1), and a heterozygous deletion of exon 4–6 in the complement factor H related gene 3 (CFHR3).

### Treatment of atypical hemolytic uremic syndrome and chronic kidney disease

As the case dates back several years, daily plasmapheresis had been started (i.e., plasma exchange of 1.0 plasma volume every day), resulting in attenuation of the hemolytic anemia whereas renal function did not recover. Nowadays administration of eculizumab may be considered [3–7]. Hemodialysis treatment was continued until 20 months later when she received a crossmatch negative AB0-compatible, nonrelated living donor kidney transplant. The immunosuppressive regimen included basiliximab, tacrolimus, and mycophenolate mofetil, and immediate transplant function was unremarkable. However, rising plasma creatinine levels were observed after transplantation together with hemolytic anemia and thrombocytopenia, indicating a relapse of atypical hemolytic uremic syndrome. One biopsy obtained 1 week after transplantation showed 17 glomeruli without thrombotic material, there were no signs for rejection, g0-1v0i1-3 t0-1ah0ptc0. Another biopsy obtained 6 weeks after transplantation showed 7 glomeruli without thrombotic material. Vessels showed increased wall thickening without thrombotic material, and immunofluorescence was positive for C3 and C4d. There were no signs for rejection, g1v0i1t0ah1ptc0.

Although tacrolimus was discontinued, whereas prednisolone, plasmapheresis, and eculizumab were started, we observed a progressive deterioration of transplant function and three months later hemodialysis treatment was resumed because of uremic symptoms.

### Treatment with eculizumab during pregnancy

The patient performed home dialysis 6 days per week for 5 h using a biocompatible membrane. Ten months later, she got pregnant. At gestational age 11 + 1 a relapse of the hemolytic anemia and thrombocytopenia was observed (hemoglobin 4.8 mmol/L, haptoglobine levels less than 0.08 g/L, platelet count 83 per nL). Intravenous eculizumab (1200 mg every other week) was started and given throughout pregnancy. The pregnancy was followed closely with repeated ultrasound monitoring growth and fetal blood flows. Intrauterine growth retardation was diagnosed due to suspected fetal distress, a healthy male index baby was delivered by cesarean section in week 34 + 2. An eculizumab infusion was given 2 h before cesarean section. Hemodialysis and eculizumab treatment were continued in the mother and follow ups in both baby and mother after 12 months were uneventful.

### Complement C3 deposition is not affected by eculizumab

**Fig. 1:**
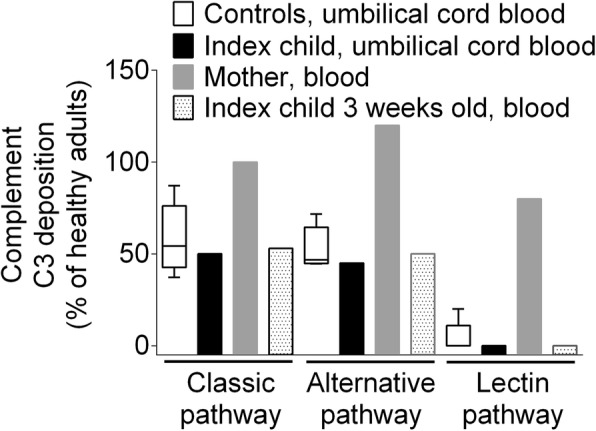
Complement C3 deposition in index newborn and mother. Complement C3 deposition after activation of the classic, alternative, and lectin pathway in umbilical cord blood from preterm new born controls (box and whiskers plot), umbilical cord blood from the index newborn, in blood from the mother treated with eculizumab, and in blood from the index newborn at 3 weeks. Results are given for the deposition of complement C3 using the Palarasah-Nielsen-ELISA [9]

In the mother’s blood, in index newborn’s umbilical cord vein blood (obtained after delivery, i.e., 2 h after the last eculizumab infusion), and in blood three weeks after birth we measured deposition of complement C3 and C9 using the Palarasah-Nielsen-ELISA as previously described [8, 9]. Fig. 1 shows the capacities of three complement pathways as determined by complement C3 deposition in the Palarasah-Nielsen-ELISA. Complement C3 deposition was similar in umbilical cord blood from control newborns and index child. The control group consisted of five pre-term (born in gestational week 35–36) boys born to healthy mothers. The lectin pathway activity was abrogated in the index baby as well as the control newborns.

### Eculizumab reduces membrane attack complex formation in the index newborn

**Fig. 2:**
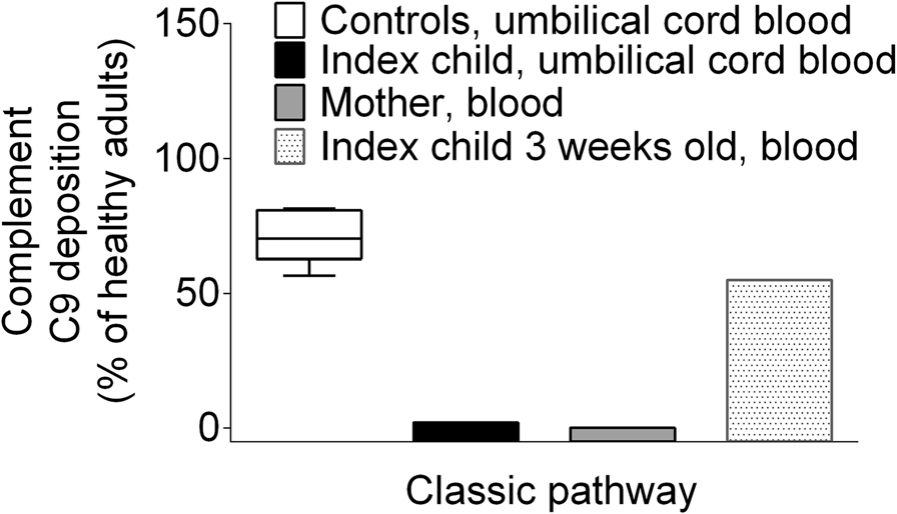
Complement C9 deposition in index newborn and mother. Complement C9 deposition after activation of the classic pathway in umbilical cord blood from preterm new born controls (box and whiskers plot), umbilical cord blood from the index newborn, in blood from the mother treated with eculizumab, and in blood from the index newborn at 3 weeks. Results are given for the deposition of complement C9 using the Palarasah-Nielsen-ELISA [9]

**Fig. 3:**
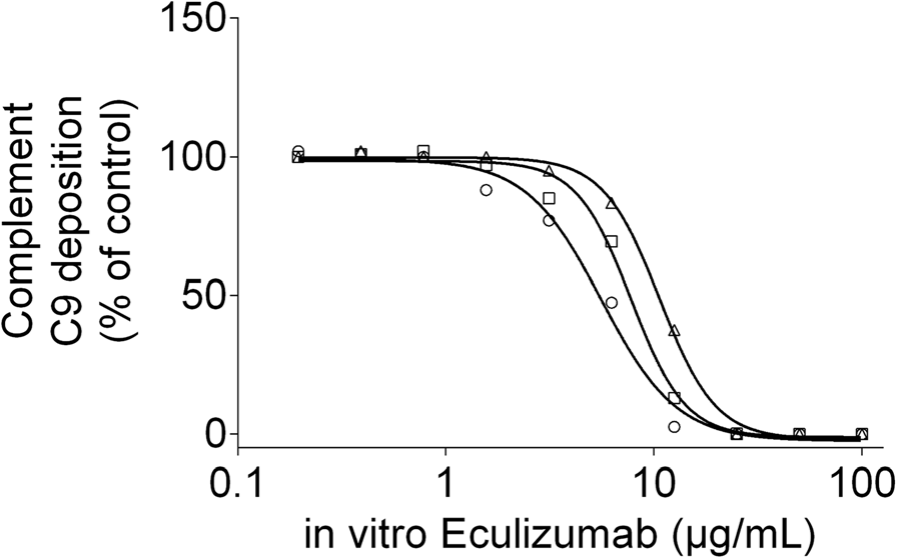
Dose-dependent effect of eculizumab on complement C9 deposition induced by activation of the classical pathway in umbilical cord blood from controls in vitro

Complement C9 deposition which occurs downstream of eculizumab inhibition is shown in Fig. 2. As expected in the mother’s blood, complement C9 deposition induced by activation of the classical pathway was completely abolished (0% compared to 59 to 130% in healthy adults) [9]. It should be noted that complement C9 deposition induced by activation of the classical pathway was almost completely abrogated in umbilical cord blood from the index newborn (2%), whereas newborn controls showed a median of 70%. The control group consisted of five pre-term (born in gestational week 35–36) boys born to healthy mothers. Complement C9 deposition normalized in the index child after 3 weeks. Furthermore, in vitro administration of 100 μg/mL complement factor C5 increased complement C9 deposition in index child from 2 to 38%. The in vitro effect of eculizumab on complement C9 deposition is depicted in Fig. 3. In-vitro administration of eculizumab to control umbilical cord blood dose-dependently reduced complement C9 deposition with apparent IC50s ranging from 6 to 10 μg/mL.

## Discussion and conclusions

Servais et al. indicated that the administration of eculizumab during pregnancy in three patients with atypical haemolytic uremic syndrome displayed no overt safety issues [10]. However, it should be noted that all babies were born preterm [10]. The index child presented in our study was also born preterm. End-stage renal disease and dialysis may well explain premature birth in our case. However, Segura-Cervantes et al. showed that women with premature preterm rupture of membranes as well as preterm labor had lower soluble C5b-9 complement complexes compared to women during term labor [11]. Human IgG are known to cross the human placental barrier especially in the third trimester [12]. Furthermore, in umbilical cord blood from three mothers with paroxysmal nocturnal hemoglobinuria or antiphospholipid syndrome eculizumab concentrations had been reported [13]. Thus eculizumab may potentially cause terminal complement inhibition in the fetal circulation [14]. This assumption is supported by our present observation. We showed that eculizumab specifically reduces complement C9 deposition, but not complement C3 deposition, in umbilical cord blood from a mother with end-stage renal disease. The impact of eculizumab was supported by the observation that complement C9 deposition could be rescued in-vitro by administration of complement C5. Furthermore, we confirmed that eculizumab may reduce formation of membrane-attack complex in umbilical cord blood from controls in-vitro. The in-vitro effect of antibodies which neutralize complement factors have recently been reported [15]. The fact that eculizumab infusion was given very close to cesarean section may explain why the present results may differ from other cases in the literature.

Genetic studies showed deletions in the gene for CFHR1 and CFHR3 in the mother. According to literature, deletions in CFHR1 and CFHR3 may be associated with atypical hemolytic uremic syndrome [2]. Our case shows that the lectin pathway activity was abrogated in the index baby as well as the control newborns, which is often found in premature children, thus excluding major contamination with blood from the mother, who had normal lectin pathway activity. The present case shows that complement C9 deposition induced by activation of the classical pathway was almost completely abrogated in umbilical cord blood from the index newborn, whereas newborn controls showed a median of 70%. These findings may indicate that the observed effect in the index newborn is more likely due to eculizumab. That is also supported by our finding that complement C9 deposition normalized in the index child after 3 weeks consistent with eculizumab’s half-life being of 12–14 days [16].

### Limitation of the study

The present study has several limitations: Additional spectrophotometric assays may be appropriate to confirm the complement findings. Furthermore, genetic findings were not based on next-gen sequencing. Another limitation is that eculizumab titers would have been helpful to strengthen the conclusions.

Taken together we give evidence that eculizumab treatment of the index child’s mother reduces the membrane-attack-complex formation in the newborn. This may cause reduced innate immunity which could render newborns more susceptible to infections.

## Data Availability

All data generated or analyzed during this study are included in this published article.

## Abbreviations

aHUS: Atypical hemolytic uremic syndrome
CFHR1: Complement factor H related gene 1
CFHR3: Complement factor H related gene 3
